# Enhanced productivity of extracellular free fatty acids by gene disruptions of acyl-ACP synthetase and S-layer protein in *Synechocystis* sp. PCC 6803

**DOI:** 10.1186/s13068-022-02197-9

**Published:** 2022-09-24

**Authors:** Kamonchanock Eungrasamee, Peter Lindblad, Saowarath Jantaro

**Affiliations:** 1 Laboratory of Cyanobacterial Biotechnology, Department of Biochemistry, Faculty of Science, Chulalongkorn University, Bangkok, 10330 Thailand; 2 Microbial Chemistry, Department of Chemistry-Ångström, Uppsala University, 523, 75120 Uppsala, Sweden

**Keywords:** Free fatty acid secretion, *Synechocystis* sp. PCC 6803, S-layer protein, Acyl–acyl carrier protein synthetase, Nitrogen deprivation

## Abstract

**Background:**

Based on known metabolic response to excess free fatty acid (FFA) products, cyanobacterium *Synechocystis* sp. PCC 6803 preferentially both recycles via FFA recycling process and secrets them into medium. Engineered cyanobacteria with well growth and highly secreted FFA capability are considered best resources for biofuel production and sustainable biotechnology. In this study, to achieve the higher FFA secretion goal, we successfully constructs *Synechocystis* sp. PCC 6803 mutants disrupting genes related to FFA recycling reaction (*aas* gene encoding acyl–acyl carrier protein synthetase), and surface layer protein (encoded by *sll1951*).

**Results:**

Three *Synechocystis* sp. PCC 6803 engineered strains, including two single mutants lacking *aas* (KA) and *sll1951* (KS), and one double mutant lacking both *aas* and *sll1951* (KAS), significantly secreted FFAs higher than that of wild type (WT). Certain increase of secreted FFAs was noted when cells were exposed to nitrogen-deficient conditions, BG_11_-half N and BG_11_-N conditions, with the exception of strain KS. Under BG_11_-N condition at day 10, strain KAS strikingly secreted FFAs products up to 40%w/DCW or 238.1 mg/L, with trace amounts of PHB. Unexpectedly, strain KS, with S-layer disruption, appeared to have endured longer in BG_11_-N growth medium. This strain KS significantly acclimated to the BG_11_-N environment by accumulating a greater glycogen pool with lower FFA production, whereas strain KA favored higher PHB and intracellular lipid accumulations with moderate FFA secretion.

**Conclusions:**

Mutations of both *aas* and *sll1951* genes in *Synechocystis* sp. PCC 6803 significantly improved the productivity of secreted FFAs, especially under nitrogen deprivation.

## Background

Despite the fact that biofuels presently are more expensive than fossil fuels, their production is growing at an exponential rate across the world. The biotechnological use of cyanobacteria for biofuel production has been classified as third and fourth generations of bioresources generating products, such as biodiesel, alka(e)ne, polyhydroxybutyrate (PHB), fatty alcohols, and energy-containing biomolecules of fatty acids and lipids [1–4]. In the field of biofuel biotechnology, the capacity of cyanobacteria to secrete free fatty acids (FFA) into the growth medium has shown to be useful in omitting the biofuel extraction process. Known strategies to enhance FFA secretion in cyanobacteria and green algae involves stressed environment effect, such as osmotic pressure, temperature, pH, and deprived nutrients, or genetically metabolic engineering, or a combination of the two [5–9]. In cyanobacteria, the cellular response mechanisms to FFAs toxicity as a result of accumulations are FFA secretion, FFA recycling, storage [9], and FFA degradation found in yeast and bacteria [10–12]. Genetically modified cyanobacteria with increased FFA secretion have been mainly observed when overexpressing genes related to thioesterase (*tesA*), catalyzing the conversion of fatty acyl–acyl carrier protein (ACP) to FFA [13], or lipase A (*lipA*), catalyzing membrane lipid degradation [9] as well as when disrupting *aas* encoding fatty acyl-ACP synthetase [9, 14, 15]. On the other hand, weakening cell walls of *Synechocystis* 6803 resulted in increased FFA secretion by disturbing genes related to the surface protein S-layer and the peptidoglycan assembly protein, PBP2 [13].

**Fig. 1:**
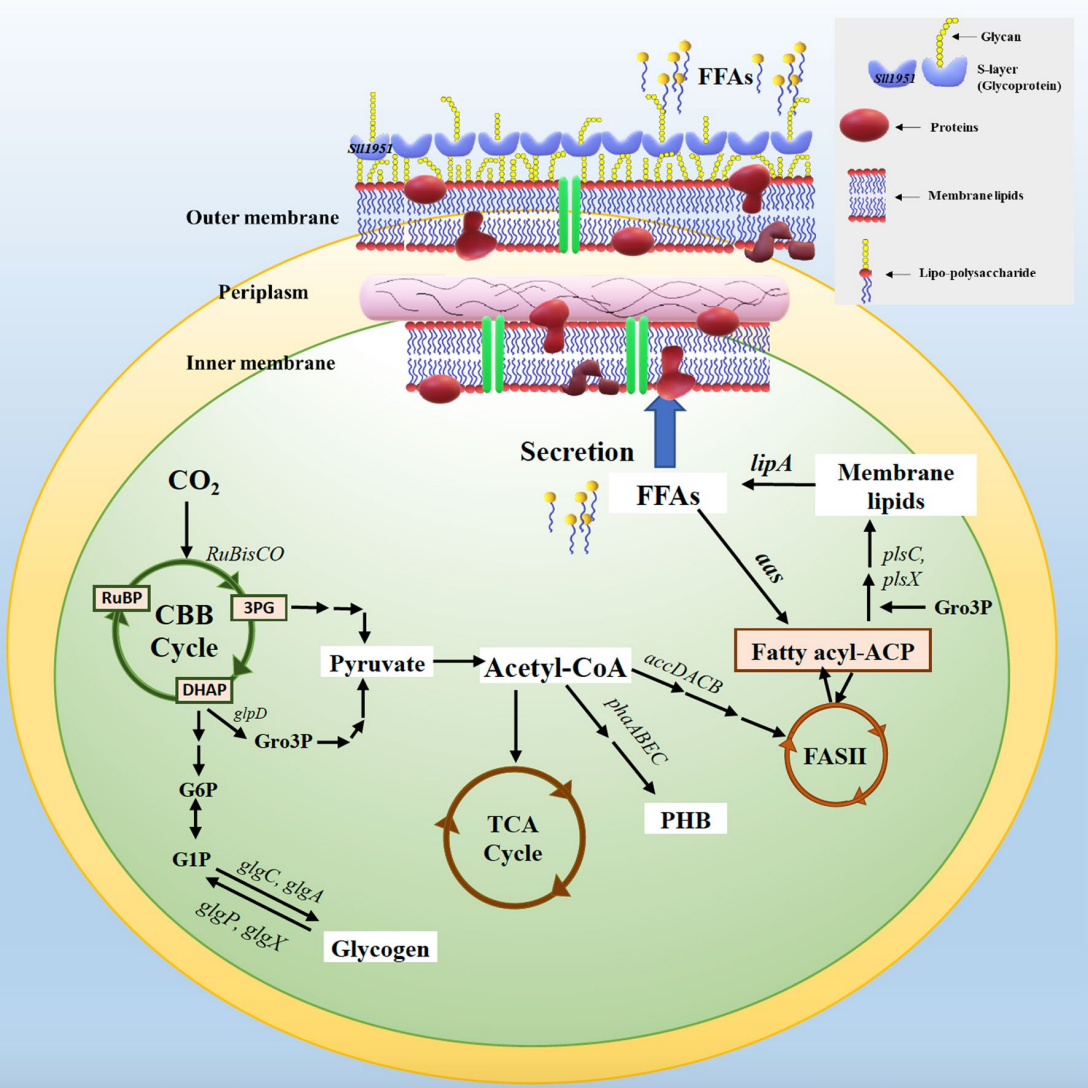
Overview of the production of lipids and free fatty acid (FFA), and FFA secretion into the growth medium in the cyanobacterium *Synechocystis* sp. PCC 6803 (modified from [9, 36]). Abbreviations of genes: *accDACB*, a multisubunit acetyl-CoA carboxylase gene; *aas*, acyl-ACP synthetase; *glgA*, glycogen synthase; *glgC*, ADP-glucose pyrophosphorylase; *glgP*, glycogen phosphorylase; *glgX*, glycogen isoamylase; *glpD*, glycerol-3-phosphate dehydrogenase; *lipA*, a lipolytic enzyme-encoding gene; *phaA*, β-ketothiolase; *phaB*, acetoacetyl-CoA reductase; *phaEC,* the heterodimeric PHB synthase; *plsX* and *plsC*, putative phosphate acyl-transferases; *RuBisCO*, the RuBisCO gene cluster including *rbcLSX*, encoding RuBisCo large, small and chaperone subunits, respectively; *sll1951*, the surface (S) layer protein. Abbreviations of intermediates: DHAP, dihydroxyacetone phosphate; FASII, fatty acid synthesis type II; fatty acyl-ACP, fatty acyl–acyl carrier protein; FFAs, free fatty acids; G1P, glucose 1-phosphate; G6P, glucose 6-phosphate; Gro3P, glycerol-3-phosphate; 3PG, 3-phosphoglycerate; PHB, polyhydroxybutyrate; RuBP, ribulose-1,5-bisphosphate; TCA cycle, Tricarboxylic acid cycle

In cyanobacteria, the main substrate for FFA production is acetyl-CoA, a pyruvate intermediate, which is further converted in various pathways, such as the TCA cycle, polyhydroxybutyrate (PHB) synthesis, and fatty acid synthesis via FASII, see Fig. 1. The fatty acyl-ACP intermediate from the FASII system is converted to membrane lipids by phosphotransacylase-type enzymes PlsX (*slr1560*), PlsC (*sll1848*), and PlsY [6, 13, 16]. For membrane lipid hydrolysis, the lipase A enzyme, encoded by *lipA* (*sll1969*), is capable of releasing free fatty acids inside the cells [3, 8, 9, 17]. The FFAs recycling to fatty acyl-ACP occurs via a fatty acyl-ACP synthetase, encoded by *aas* (*slr1609*) [3]. Moreover, excess of FFAs may be secreted by rapidly flip–flopping the un-ionized form of FFA through protein channels of membranes, such as efflux transmembrane transporters (*sll0180* and *slr2131*) [18, 19]. For the surface layer (S-layer) on cell walls of cyanobacteria, its disruption results in increased FFA secretion [13]. The functions of S-layer proteins are mainly involved in carbon capture and storage (CCS) and CO_2_ diffusion through the cell membranes in relation to bicarbonate (HCO_3_^−^) in *Synechocystis* sp. PCC6803 [20]. This S-layer protein has a supportive role for cell wall integrity in *Synechocystis* without any lethal effect in a *Δsll1951* strain [21]. The carbon storage form of glycogen, glycogen is synthesized from glucose-1-phosphate (G1P) and ADP-glucose intermediates via glucose-1-phosphate adenylyltransferase (*glgC*) and glycogen synthase (*glgA1* and *glgA2*), respectively, whereas its degradation is catalyzed by glycogen phosphorylase (*glgP*) and isoamylase (*glgX*) [22]. Under nitrogen deficiency condition, the glycogen pool may eventually be degraded to produce the other carbon storage form polyhydoxybutyrate (PHB) [22, 23]. To cope with environmental stresses with induced cells accumulating energy storage, the cyanobacterial PHB is preferentially produced from acetyl-CoA through multiple enzymes including acetyle-CoA acetyltransferase (*phaA*), acetoacetyl-CoA reductase (*phaB*)*,* and the heterodimeric PHB synthase (*phaEC*) [24–27].

In this study, we successfully created genetically engineered *Synechocystis* sp. PCC 6803 strains with high production of FFAs secreted into the growth medium using knockout (K) mutations of *aas* (A) and/or *sll1951* (S), genes encoding fatty acyl-ACP synthetase and S-layer protein, respectively, resulting in strains KA, KS, and KAS. We discovered that a considerable long-term adaptation of the KS strain to nitrogen deprivation (BG_11_-N) resulted in increased glycogen storage with a comparable PHB pool and decreased FFA production when compared to *Synechocystis* sp. PCC 6803 wild type (WT) cells. Interestingly, the double mutant of the KAS strain released at least 5 times more FFAs than wild type cells while having the lowest PHB accumulation during nitrogen deprivation. The KA strain accumulated more intracellular lipids than the KAS strain, but secreted less FFA. Among all strains investigated, the KA strain showed the highest level of PHB under BG_11_-N condition.

## Results

### Synechocystis sp. PCC 6803 engineered strains and their growth under stress conditions

**Fig. 2:**
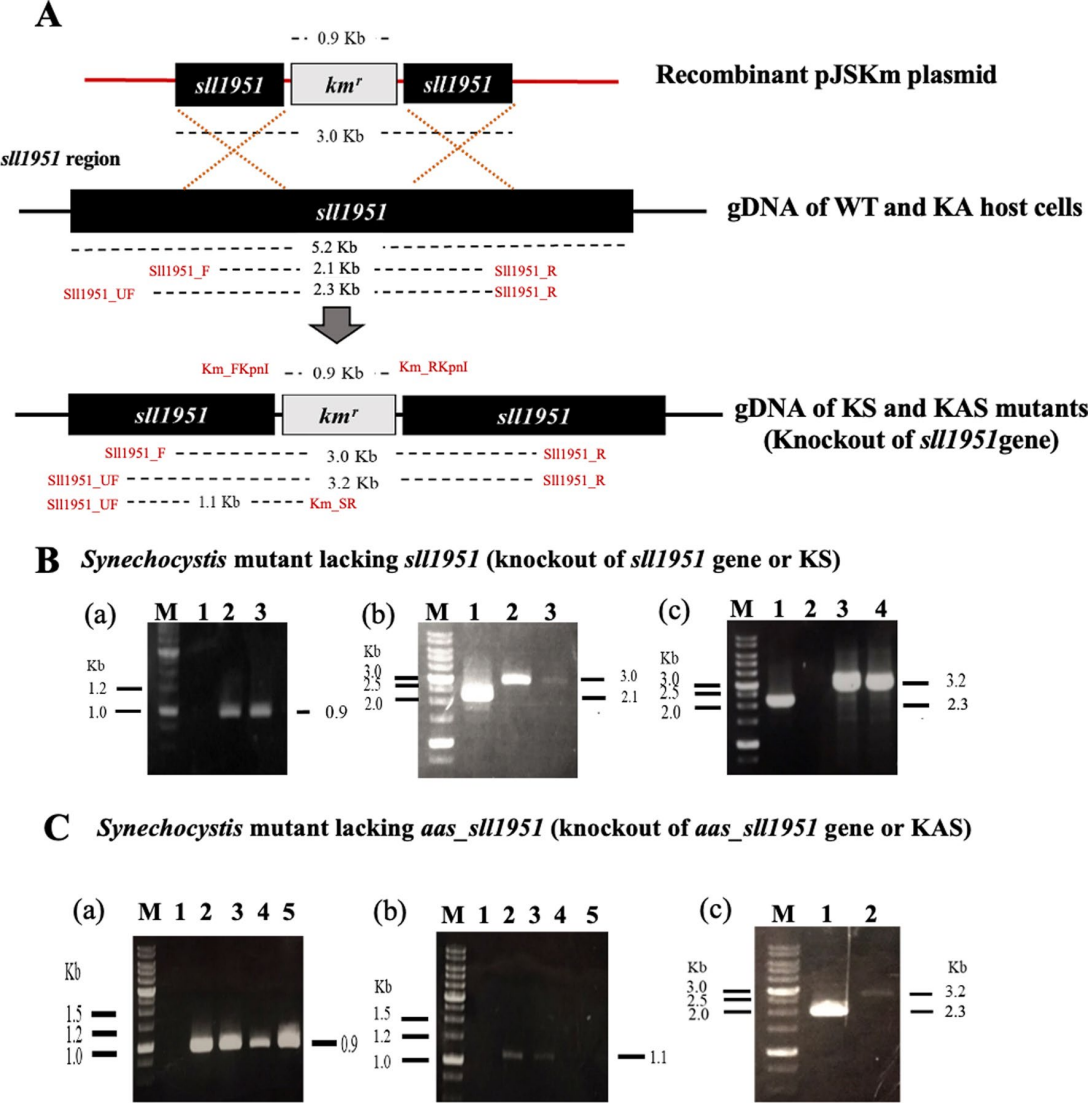
Genomic maps of the engineered *Synechocystis* sp. PCC 6803 strains, KS and KAS. **A** Double homologous recombination occurred between the conserved sequences of *sll1951* or *S-layer* gene on the recombinant pJSKm plasmid containing an antibiotic kanamycin resistant cassette (*Km*^*r*^) and genomic DNA of WT or KA host strain, generating KS or KAS strain, respectively. Confirmations of engineered strains were performed by PCR analysis using selected pairs of specific primers (shown in Table 2). For (**B**) KS strain; Lane M: GeneRuler DNA ladder (Fermentus); Lane 1: Negative control using WT as template (a–c), a Lanes 2–3: clone numbers 1 to 2 using Km_F and Km_R primers, b Lanes 2–3: clone numbers 1 to 2 using Sll1951_F and Sll1951_R primers, and c Lanes 2–4: clone numbers 1 to 3 using Sll1951_UF and Sll1951_R primers, and only positive clones (numbers 2 and 3) were selected for next experiments. For (**C**) KAS strain. Lane M: GeneRuler DNA ladder (Fermentus); Lane 1: Negative control using KA as template (a–c), a Lanes 2–5: clone numbers 1 to 4 using Km_F and Km_R primers, b Lanes 2–5: clone numbers 1 to 4 using Sll1951_UF and Km_SR primers, and c Lane 2: clone number 1 using Sll1951_UF and Sll1951_R as the primers, and only positive clone number 1 was selected for next experimentsq4

First, the *sll1951* gene of WT and KA strains (Table 1) was disrupted through the integral insertion of a 0.9 kb fragment of a kanamycin cassette gene (*km*^*r*^) to generate a knockout of *sll1951* (KS) and a knockout of *aas/sll1951* (KAS) strains (Fig. 2A). To confirm the segregation and location of the insertions (Fig. 2B, C), PCRs using gDNA of each strain as template and selected specific primers were performed (Table 2). Both strains KS and KAS contained the *km*^*r*^ fragment with a size of about 0.9 Kb, compared to those of WT and KA with *km*^*r*^ fragment, Fig. 2B-a, C-a. In addition, PCR products with Sll1951_F and Sll1951_R primers confirmed the correct size of 3.0 Kb in strain KS, whereas it was 2.1 Kb in WT (Fig. 2B-b). The Sll1951_UF and Km_SR primers confirmed the expected size of about 1.1 Kb in strain KAS comparing with no band in WT (Fig. 2C-b). When we amplified the fragment by primers Sll1951_UF and Sll1951_R, the PCR products gave a 3.2 Kb band in both KS and KAS strains, while it showed a 2.3 Kb band in the WT (Fig. 2B-c, C-c).

**Table 1:** Strains and plasmids used in this study

Name	Relevant genotype	Reference
Cyanobacterial strains		
*Synechocystis* sp. PCC 6803	Wild type	Pasteur culture collection
Control WT (WTc)	*cm*^*r*^ and *km*^*r*^ integrated at region of native *psbA2* gene in *Synechocystis* genome	[9]
KA	*cm*^*r*^ integrated at region of native *aas* gene in *Synechocystis* genome	[9]
KAOL	*cm*^*r*^ integrated at region of native *aas* gene in *Synechocystis* genome *lipA, km*^*r*^ integrated at region of native *psbA2* gene in *Synechocystis* genome	[9]
KS	*km*^*r*^ integrated at region of native *sll1951* gene in *Synechocystis* genome *glpD, Rubisco; rbcL,rbcX, rbcS, km*^*r*^ integrated at region of native *Rubisco* gene in *Synechocystis* genome	This study
KAS	*km*^*r*^ integrated at region of native *sll1951* gene in *Synechocystis* genome *cm*^*r*^ integrated at region of native *aas* gene in *Synechocystis* genome	This study
*Plasmids*		
pJSKm	P_T7_–*sll1951-cm*^*r*^; plasmid containing *km*^*r*^ between the flanking region of *sll1951* gene	This study

**Table 2:** Primers used in this study

Name	Sequence (5ʹ to 3ʹ)	Purpose of primer	Expected size	Cycles/Tm	Reference
Km_FKpnI	TAGAGAGGTACCTTAGAAAAACTCATCGAG CA	PCR for *km*^*r*^	939	30/60.0 °C	This study
Km_RKpnI	TAGAGAGGTACCGTGTCTCAAAATCTCTGATG	PCR for *km*^*r*^			This study
Km_SR	TAGAGATCAGTCGTCACTCATGGTGA	PCR for *km*^*r*^			This study
Sll1951_F	TAGAGAGTGGAAGATGCAAAT ATACT	PCR for *sll1951*	1980	35/56.3 °C	This study
Sll1951_R	TAGAGAGGCGCTATCACT GGTAAAAG	PCR for *sll1951*			This study
Sll1951_UF	TAGAGAGTGGAAATTGCG GCTTCC CT	PCR for *sll1951*			This study
RTglgX_F360	GAGCTTCATCGAGGACGGAA	RT-PCR for *glgX*	360	35/56.0 °C (BG_11_)	This study
RTglgX_R360	GCCCGAATTGGGGTTGCGGG	RT-PCR for *glgX*		30/56.0 °C (BG_11_-N)	
RTphaA_F420	TCAGCCGGATAGAATTGGACG AAGT	RT-PCR for *phaA*	420	35/53.5 °C (BG_11_)	[8]
RTphaA_R420	CAAACAAGTCAAAATCTGCCA GGGTT	RT-PCR for *phaA*		30/53.5 °C (BG_11_-N)	
RTlipA_F379	TTGGCGGAGCAAGTGAAGCAAT	RT-PCR for *lipA*	379	34/55.0 °C (BG_11_)	[8]
RTLipA_R379	CATGGACCAGCACAGGCAAAAT	RT-PCR for *lipA*		28/55.0 °C (BG_11_-N)	
RTaccA_F428	ATGCACGGCGATCGAGGAGGT	RT-PCR for *accA*	428	35/58.0 °C (BG_11_)	[8]
RTaccA_R428	TGGAGTAGCCACGGTGTACAC	RT-PCR for *accA*		32/58.0 °C (BG_11_-N)	
RT16sRNA_F521	AGTTCTGACGGTACCTGATGA	RT-PCR for *16 s*	521	24/56.0 °C (BG_11_)	[8]
RT16sRNA_R521	GTCAAGCCTTGGTAAGGTTAT	RT-PCR for *16 s*		22/56.0 °C (BG_11_-N)	
RTaas_F307	GTGGTTTATCGCCGATCAAG	RT-PCR for *aas*	307	38/54.5 °C (BG_11_)	[8]
RTaas_R307	TTCCTGGCGGGGAACGGGAG	RT-PCR for *aas*		33/54.5 °C (BG_11_-N)	
RTPlsX_F	AAGGGGTGGTGGAAATGGAA	RT-PCR for *PlsX*	488	35/52.7 °C (BG_11_)	[6]
RTPlsX_R	AAGTAGGTCCCTTCCTTCGG	RT-PCR for *PlsX*		32/52.7 °C (BG_11_-N)	

**Fig. 3:**
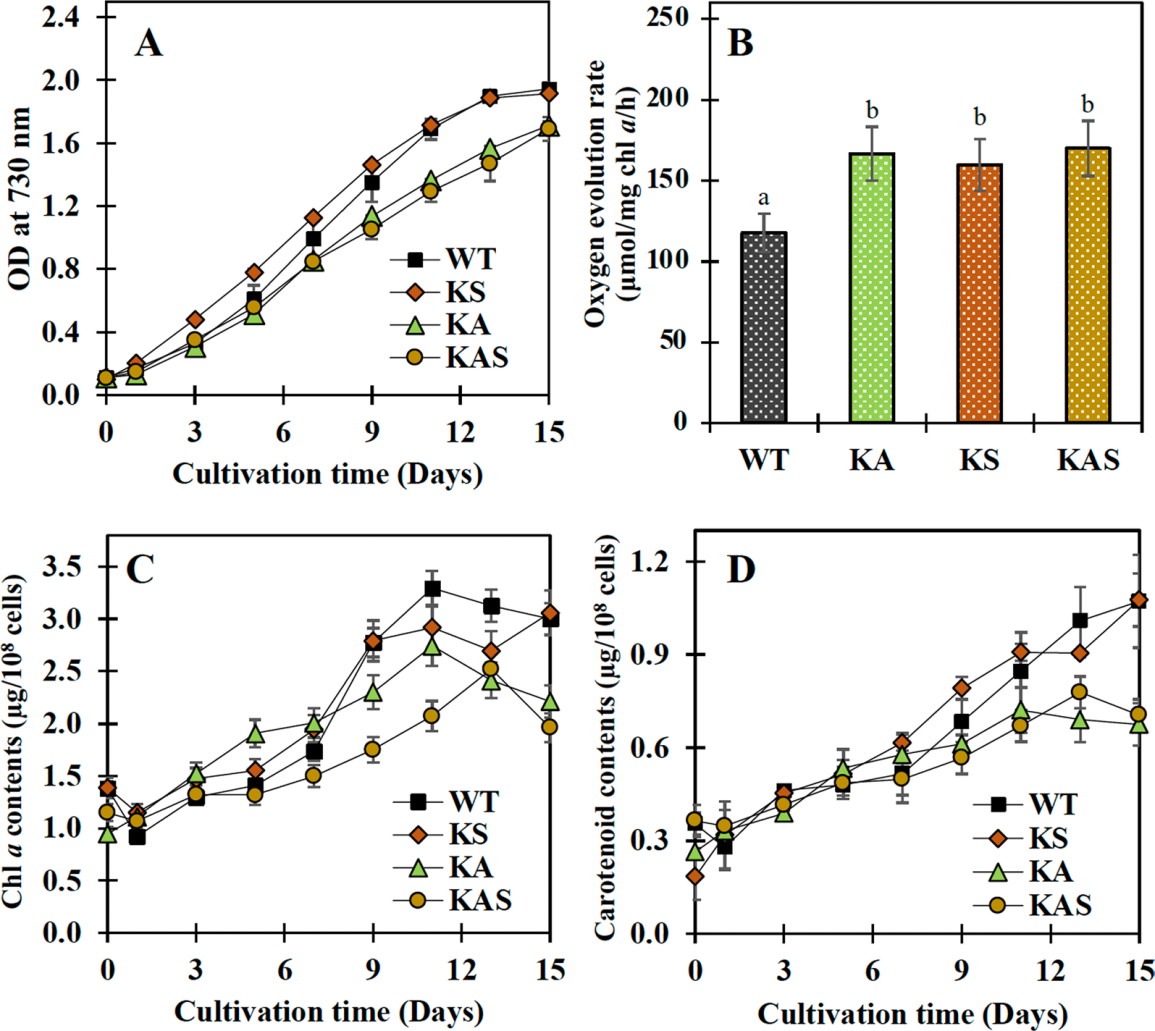
Growth curve (**A**), oxygen evolution rate (**B**), chlorophyll *a* content (**C**), and carotenoid content (**D**) of WT, KS, KA, and KAS *Synechocystis* sp. PCC 6803 strains cultured in BG_11_ medium for 14 days. In (**A**), (**C**), and (**D**), the error bars represent standard deviations of means (mean ± S.D., *n* = 3). In (**B**), the oxygen evolution rate was measured using log phase-growing cells (5 days). Data represent mean ± S.D., *n* = 3. Means with the same letter are not significantly different with the significance level at *P* < 0.05

**Fig. 4:**
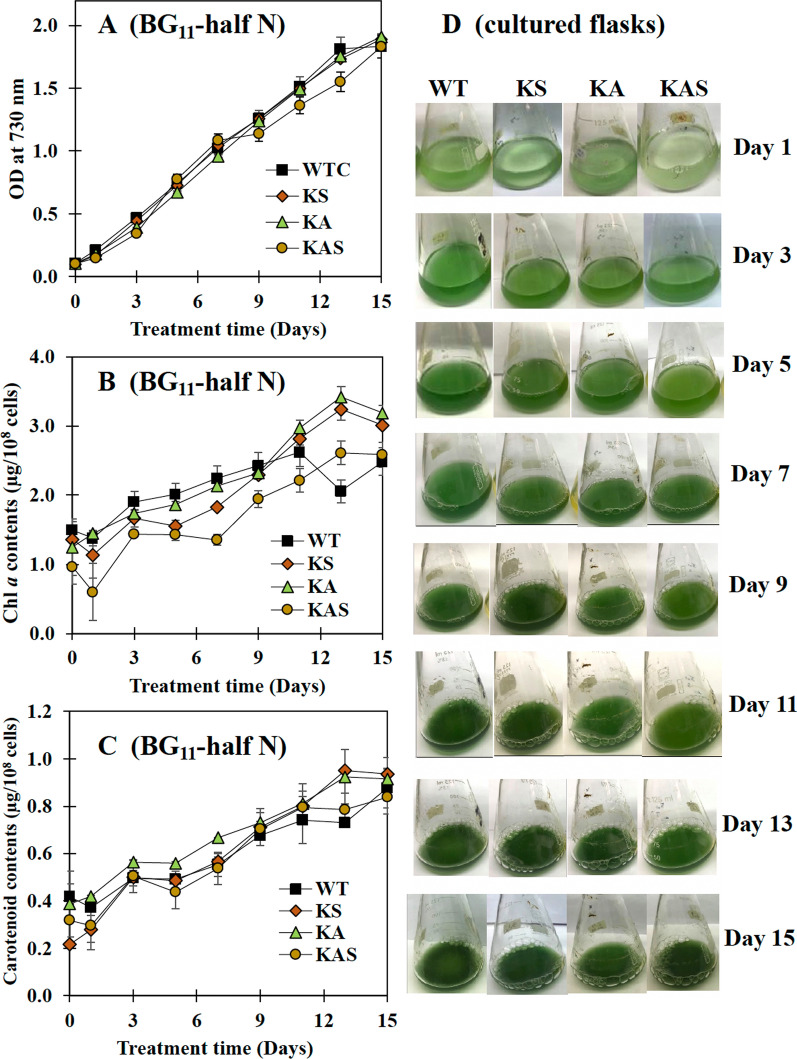
Growth curve (**A**), chlorophyll *a* content (**B**), carotenoid content (**C**), and images of cultured flasks of WT, KS, KA, and KAS *Synechocystis* sp. PCC 6803 strains cultured in BG_11_ containing 8.8 mM NaNO_3_ medium (BG_11_-half N) during 15 days of cultivation. The error bars represent standard deviations of means (mean ± S.D., *n* = 3)

**Fig. 5:**
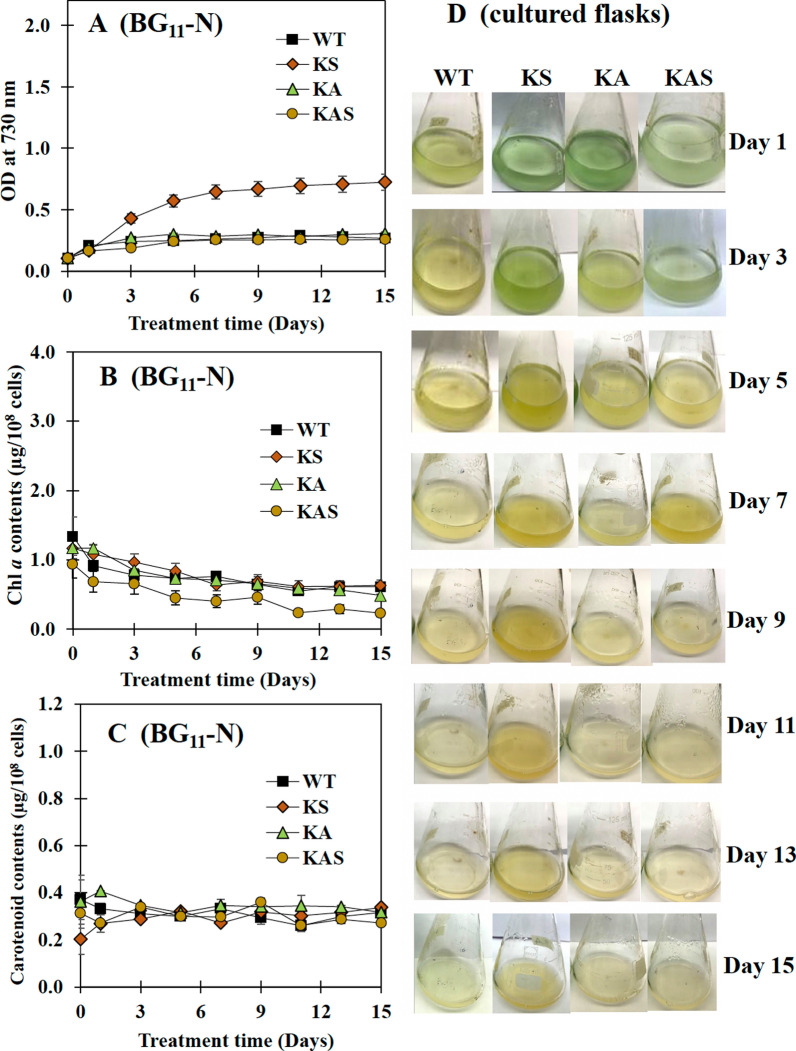
Growth curve (**A**), chlorophyll *a* content (**B**), carotenoid content (**C**), and images of cultured flasks of WT, KS, KA, and KAS *Synechocystis* sp. PCC 6803 strains cultured in BG_11_ without the addition of NaNO_3_ medium (BG_11_-N) during 15 days of cultivation. The error bars represent standard deviations of means (mean ± S.D., *n* = 3)

Cell growths of the KA and KAS strains were lower than that of the wild type (WT) cells under BG_11_ growth conditions, although the KS strain exhibited a similar tendency as WT (Fig. 3A). It was intriguing to see that the oxygen evolution rates of all engineered strains were significantly higher than those of WT cells (Fig. 3B). Furthermore, the KS strain accumulated equivalent levels of chlorophyll *a* and carotenoids as the WT strain (Fig. 3C, D). The KA and KAS strains showed lower quantities of chlorophyll *a* and carotenoids, in agreement with their respective growth. On the other hand, all strains could grow similar to WT in BG_11_ with half concentration of NaNO_3_ (BG_11_-half N), with the exception of the KAS strain, which showed a slightly lower growth after 9 days (Fig. 4A). Under this growth condition, the KA and KS strains contained more chlorophyll *a* and carotenoids after 9 days (Fig. 4B, C). Images of cell culture in BG_11_-half N clearly demonstrated that strain KAS showed a lighter green color than the other strains (Fig. 4D), reflected in a lower chlorophyll *a* content (Fig. 4B). In line with growth and chlorophyll *a* content, KS and KA cell cultures showed a more deep green color under half N growth condition. When BG_11_ lacking NaNO_3_ condition (or BG_11_-N) was applied to all strains (Fig. 5). Strain KS showed the highest growth level (Fig. 5A). The chlorophyll *a* levels were comparable between the strains, with the exception of KAS which contained a lower amount (Fig. 5B). However, the carotenoid levels were relatively stable under BG_11_-N condition (Fig. 5C). It is clear from the images of cell cultures grown in BG_11_-N that all engineered strains remained green for at least 3 days before becoming yellow compared to WT cells, particularly strain KS (Fig. 5D). The KAS strain had a deep yellowish cell culture from days 5 to 7, whereas strain KS strain showed a deep yellowish cell culture from days 5 to 15.

### Contents of intracellular lipids, extracellular FFAs, PHB and glycogen under normal and stressed conditions

**Fig. 6:**
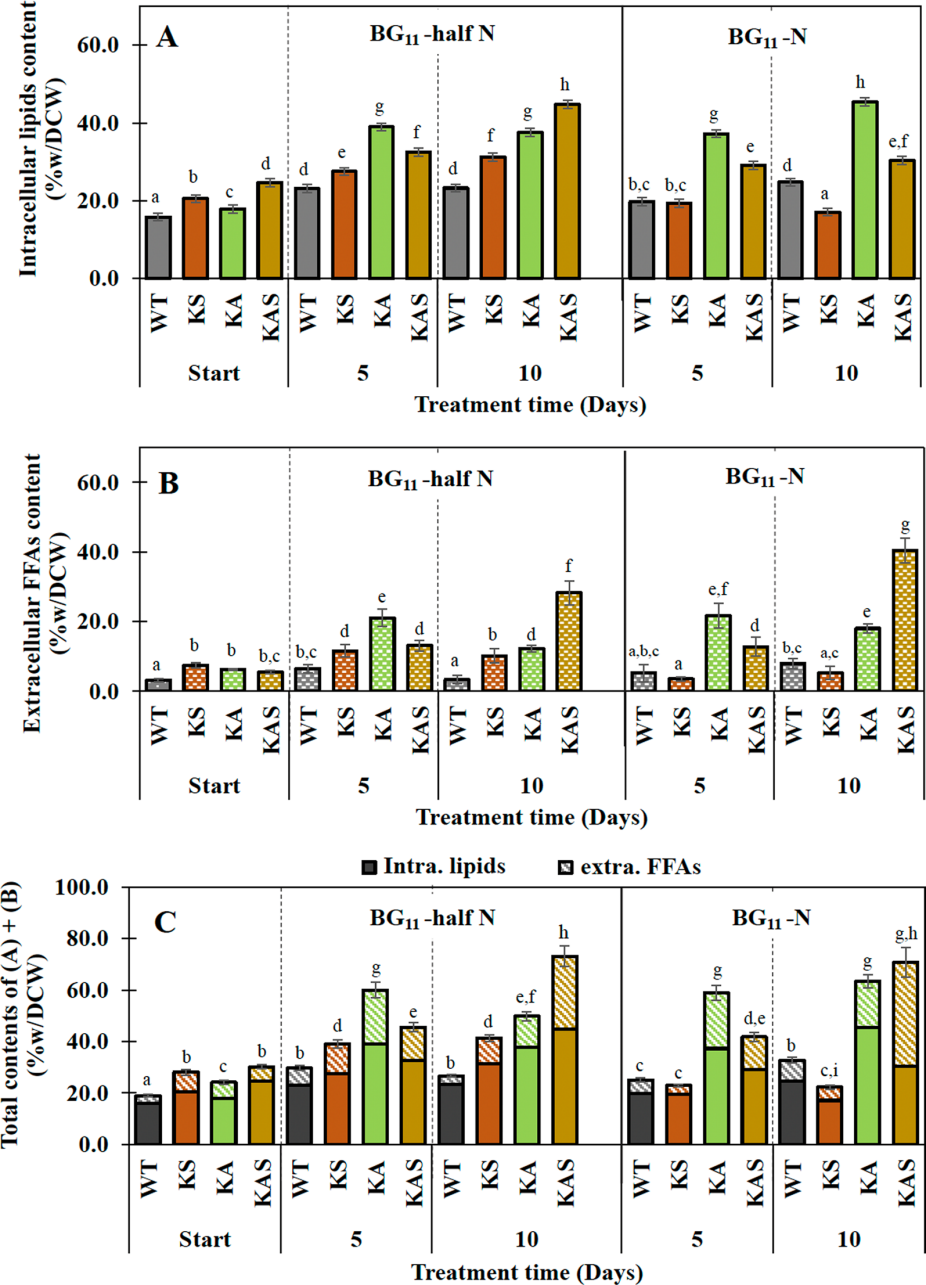
Contents of total intracellular lipids (**A**) and extracellular FFAs (**B**), and total contents of total intracellular lipids and extracellular FFAs (**C**) of WT, KS, KA, and KAS *Synechocystis* sp. PCC 6803 strains growing in BG_11_-half N and BG_11_-N at 0, 5 and 10 days, respectively. The error bars represent standard deviations of means (mean ± S.D., *n* = 3). Means with the same letter are not significantly different with the significance level at *P* < 0.05

All engineered strains secreted more FFAs into the BG_11_ growth medium, Table 3. Strains KA and KAS notably contained higher total amounts of intracellular lipids and extracellular FFAs by about 35.8 and 39.0%w/DCW, respectively, than WT cells (23.6%w/DCW), in particular at day 5. In addition, we observed that all engineered strains had certain total yields (mg/L) that were higher than WT at days 5 and 10, particularly in strain KAS produced about 178.5 and 336.9 mg/L, respectively (Table 3). After exposing the cells to reduced levels of nitrogen (BG_11_-half N), all engineered strains produced higher levels of intracellular lipids than observed in WT cells, in particular strain KA at day 5 and KAS at day 10 with 39.0 and 44.8%w/DCW, respectively (Fig. 6A). The certain increase of FFA secretion of all engineered strains was also noted under this condition with the highest level in strain KAS at day 10, about 28.2%w/DCW (Fig. 6B), representing 376.2 mg/L or 53.3 mg/10^11^ cells (Table 4). On the other hand, when the strains were grown in BG_11_-N medium, we discovered that the KS strain accumulated extracellular FFAs at the same level, either %w/DCW or mg/L, as the WT, whereas the KA and KAS strains showed increased level (Fig. 6B and Table 4). The results indicate that strain KAS preferentially secreted FFAs into medium up to 40.4%w/DCW or 238.1 mg/L after a long period (10 days) of nitrogen deprived condition (BG_11_-N) rather than accumulated intracellular lipids (30.4%w/DCW) when compared to strain KA, 45.5 and 18.0%w/DCW% of intracellular lipids and extracellular FFAs contents, respectively (Fig. 6A–C).

**Table 3:** Yields of intracellular lipids and extracellular FFAs of all strains under normal BG_11_ condition

Strains	Contents (%/DCW)			Yields (mg/L)			Note
	Intracellular lipids (A)	Extracellular FFAs (B)	Total (A) + (B)	Intracellular lipids (A)	Extracellular FFAs (B)	Total (A) + (B)	
Start of cultivation							
WT	12.4 ± 1.13^a^	1.53 ± 0.22^f^	13.9 ± 1.35^a,c^	7.69 ± 0.23^A^	0.68 ± 0.05^F^	8.37 ± 0.28^E^	This study
KS	10.9 ± 0.50^a^	4.46 ± 0.12^g^	15.4 ± 0.62^c^	5.45 ± 0.25^B^	0.89 ± 0.02^G^	6.34 ± 0.27^D^	This study
KA	17.6 ± 0.24^b^	4.52 ± 1.17^g^	22.1 ± 1.41^e^	10.6 ± 0.14^C^	1.35 ± 0.35^H^	11.9 ± 0.49^J,C^	[9]
KAS	18.1 ± 1.27^b^	7.42 ± 0.66^h^	25.5 ± 1.93^k^	10.8 ± 1.36^C^	2.22 ± 0.20^I^	13.0 ± 1.56^K,J,C^	This study
Day 5 of cultivation							
WT	16.7 ± 1.37^b,c^	6.8 ± 1.35^g,h^	23.6 ± 2.72^d,e,k^	126.9 ± 7.26^Q^	7.81 ± 1.64^A,D^	134.7 ± 8.90^Q,R^	This study
KS	17.2 ± 1.24^b,c^	11.1 ± 1.25^a^	28.3 ± 2.49^k,l^	154.2 ± 14.6^S^	15.0 ± 1.62^K^	169.2 ± 16.2^T^	This study
KA	20.6 ± 0.65^d^	15.2 ± 1.12^c^	35.8 ± 1.77^m,n^	137.4 ± 0.57^R^	21.1 ± 0.84^L^	158.5 ± 1.41^S^	[9]
KAS	23.0 ± 0.48^e^	16.0 ± 1.02^c^	39.0 ± 1.50^n^	155.8 ± 0.87^S^	22.7 ± 2.16^L^	178.5 ± 3.03^T^	This study
Day 10 of cultivation							
WT	15.5 ± 0.52^c^	5.6 ± 0.46^g^	21.1 ± 0.97^d^	143.2 ± 5.30^R,S^	31.0 ± 1.91^M^	174.2 ± 7.21^T^	This study
KS	19.2 ± 1.42^b,d^	9.1 ± 0.49^j^	28.3 ± 1.91^k,l^	177.1 ± 15.0^T^	50.1 ± 2.37^N^	227.2 ± 17.4^U^	This study
KA	16.4 ± 0.79^b,c^	13.9 ± 0.64^a^	30.3 ± 1.43^l^	255.2 ± 20.0^V,U^	108.4 ± 11.4^P^	363.6 ± 31.4^W^	[9]
KAS	16.3 ± 0.48^b,c^	12.1 ± 1.02^a^	28.4 ± 1.50^k,l^	245.2 ± 15.8^V,U^	91.7 ± 10.7^P^	336.9 ± 26.5^W^	This study

Data represent mean ± S.D., *n* = 3. For superscript, means with the different letter are significantly different with the significance level at *P* < 0.05

**Table 4:** Yields of extracellular FFAs of all strains under normal BG_11,_ BG_11_-half N, and BG_11_-N condition

Strains	Extracellular FFA titer (mg/L)			Extracellular FFA (mg/10^11^ cells)		
	Start	Day 5	Day 10	Start	Day 5	Day 10
BG_11_-half N condition						
WT	0.53 ± 0.12^a^	18.21 ± 6.10^h^	29.52 ± 5.73^k^	1.46 ± 0.19^A^	6.11 ± 1.99^D^	5.30 ± 1.09^D^
KS	1.20 ± 0.09^c^	28.81 ± 6.55^j,i^	101.43 ± 8.05^q^	2.17 ± 0.09^B^	9.93 ± 2.58^E^	17.8 ± 1.80^F^
KA	1.25 ± 0.02^d,c^	92.38 ± 19.25^m^	182.54 ± 10.80^r^	2.43 ± 0.59^B^	34.3 ± 6.80^I,H^	32.0 ± 1.05^I^
KAS	1.73 ± 0.07^e^	62.38 ± 2.18^l^	376.19 ± 63.57^t^	4.04 ± 0.28^C,D^	20.0 ± 0.64^G,F^	53.3 ± 0.46^J^
BG_11_-N condition						
WT	0.77 ± 0.08^a^	6.81 ± 1.66^g^	30.24 ± 7.33^k^	1.72 ± 0.18^A^	13.6 ± 3.01^E^	15.1 ± 3.39^F,E^
KS	1.04 ± 0.15^b^	8.24 ± 1.20^g^	32.14 ± 7.56^k^	2.54 ± 0.38^B^	7.26 ± 1.29^D^	7.11 ± 2.06^D,E^
KA	1.19 ± 0.02^c,b^	34.57 ± 4.88^k^	65.87 ± 3.64^p^	2.67 ± 0.08^B^	28.9 ± 4.78^I,H^	32.9 ± 1.72^I^
KAS	1.73 ± 0.02^e^	22.86 ± 1.78^i^	238.10 ± 36.67^s^	3.94 ± 0.13^C^	23.9 ± 1.82^H^	149 ± 22.6^K^

Data represent mean ± S.D., *n* = 3. For superscript, means with the different letter are significantly different with the significance level at *P* < 0.05

**Fig. 7:**
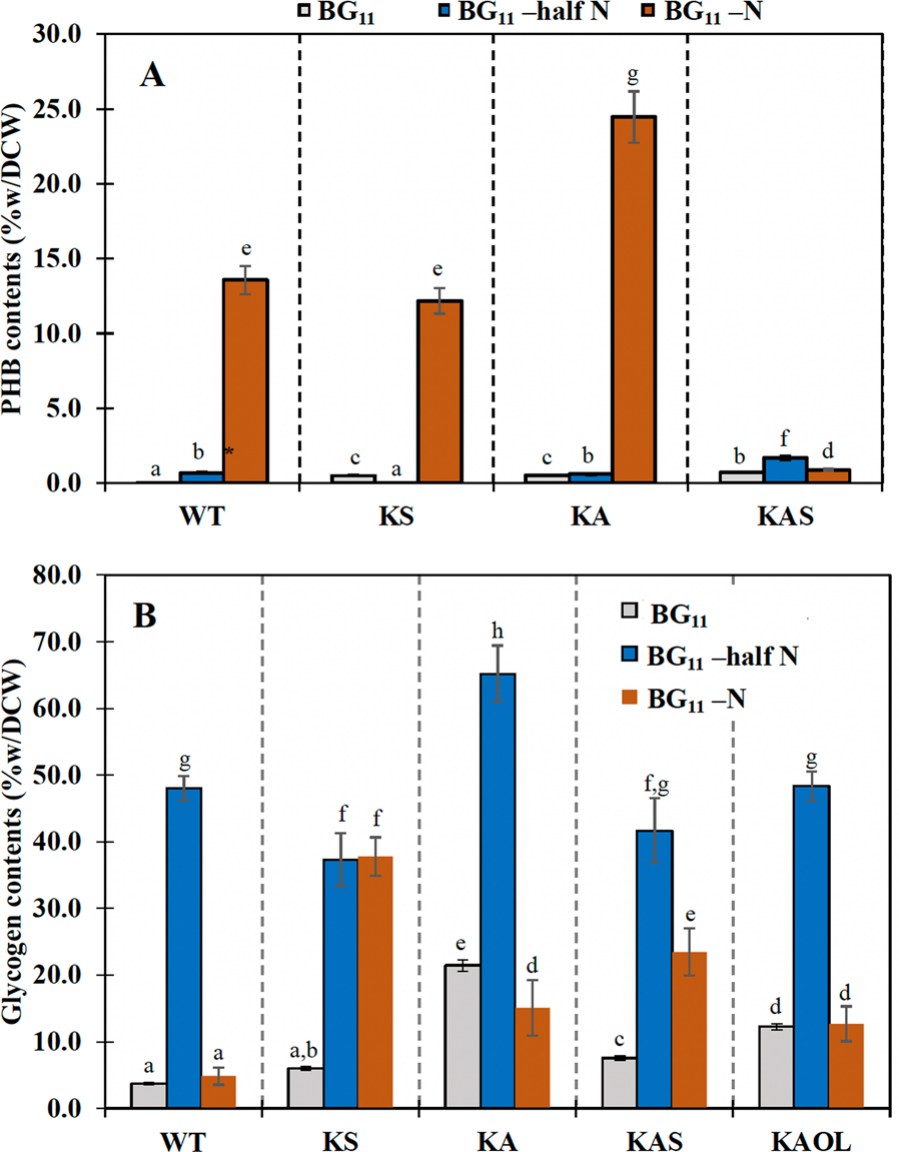
Contents of polyhydroxybutyrate (PHB) (**A**) and glycogen (**B**) of *Synechocystis* sp. PCC 6803 WT, KS, KA, KAS, and KAOL strains cultured under BG_11_-half N and BG_11_-N at day 10. The error bars represent standard deviations of means (mean ± S.D., *n* = 3). Means with the same letter are not significantly different with the significance level at *P* < 0.05

We also determined polyhydroxybutyrate or PHB contents of all strains under BG_11_, BG_11_-half N, and BG_11_-N growth conditions at day 10 (Fig. 7A). Unexpectedly, a substantial increase in PHB content occurred in all strains under BG_11_-N conditions, with the exception of strain KAS, which showed a low level equivalent to that under BG_11_ condition. Not all strains were affected by the BG_11_-half N condition, only strain KAS showed a 2.3 fold-increase in PHB accumulation when compared to WT cells. On the other hand, the glycogen content of all engineered strains were higher than in WT cells under BG_11_ condition, especially in strain KA with 21.4%w/DCW (Fig. 7B). It is worthy to note that the BG_11_-half N condition highly induced the glycogen accumulation in all strains examined. The KA strain accumulated significantly more glycogen up to 65.1%w/DCW. When the BG_11_-N condition was applied, the increased levels of glycogen were observed in strains KS and KAS, compared to under BG_11_ medium. It is interesting that strain KS showed similar glycogen content under both BG_11_-half N and BG_11_-N growth conditions.

**Fig. 8:**
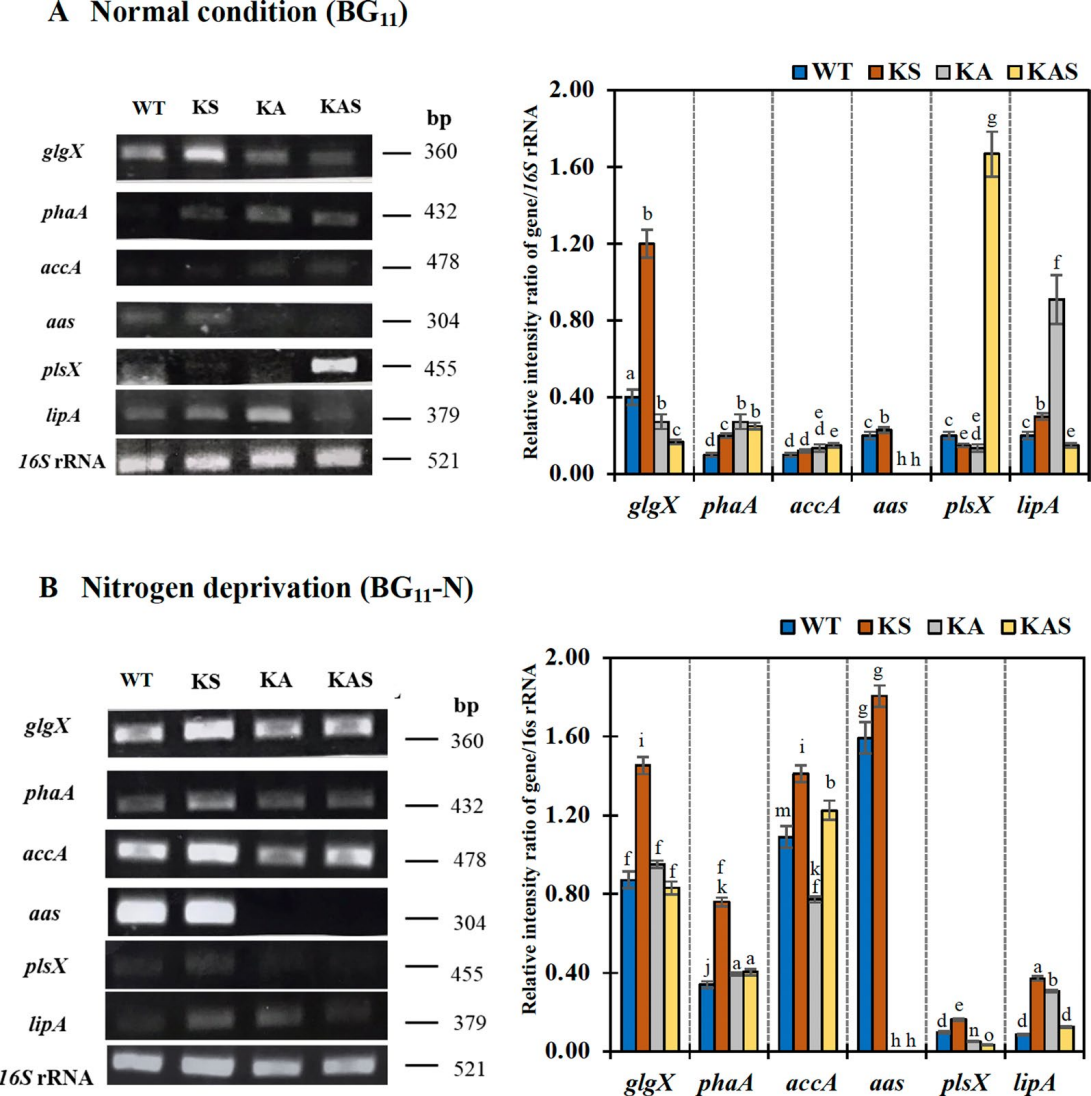
Transcript levels of genes including *glgX, phaA, accA*, *aas, plsX, lipA* and *16S rRNA* of WT, KS, KA, and KAS *Synechocystis* sp. PCC 6803 strains under BG_11_ (**A**) and BG_11_-N (**B**) conditions. Cell culture at day 10 of treatment were harvested and analyzed. On the right hand side, the relative intensity ratios of each gene/*16S* rRNA were analyzed by GelQuant.NET program. Data represent mean ± S.D., *n* = 3. Means with the same letter are not significantly different with the significance level at *P* < 0.05

Moreover, transcript levels of genes related to fatty acid synthesis, its degradation, PHB synthesis and glycogen degradation were monitored in cells at day 10 of growth (Fig. 8). Under BG_11_ condition (Fig. 8A), the *accA* transcript levels, related to the initial step of fatty acid synthesis, were slightly increased in strains KA and KAS. The *plsX* transcript level, which is related to membrane lipid synthesis, was greatly elevated in strain KAS. The *lipA* transcript levels, related to membrane lipid hydrolysis, were increased in strains KS and KA but decreased in strain KAS. For PHB synthesis, the *phaA* transcript levels were slightly upregulated in all engineered strains corresponded to higher PHB contents when compared to WT cells. The *glgX* transcript amounts, related to glycogen degradation, showed a significant upregulation in strain KS, whereas decreased levels were observed in strains KA and KAS, compared to WT cells under BG_11_ growth condition. In addition, similar *aas* transcript levels, related to FFA recycling reaction, of WT and KS cells were observed. On the other hand, the higher ratio values of transcript/16* s* band intensity of *accA, aas, phaA and glgX* in WT cells were noted under BG_11_-N condition when compared to those under BG_11_ condition, in Fig. 8B. The transcript levels of *accA*, *aas, plsX, lipA, phaA* and *glgX* in strain KS were higher than those in WT cells. For strain KA, only the *lipA* transcript level was increased, whereas similar or decreased levels were observed for the other genes.

## Discussion

To increase free fatty acid (FFA) secretion, genetically engineered cyanobacteria are considered as a promising option. However, FFAs secretion as a consequence of excessive production of FFAs may generate toxicity and damage the cells by randomly diffuse across the membranes, in particular short chain FFAs, generating reactive oxygen species (ROS) and a highly oxidative stressful environment for the cells [28–30]. Some recent reports addressed the crucial consequences associated with higher FFA secretion after modifying the cyanobacterium *Synechocystis* sp. PCC6803 by gene disruption, such as *aas, sll1951* encoding surface layer (S-layer) protein, and *slr1710* encoding peptidoglycan assembly protein, or by overexpression of heterologous *tesA* encoding thioesterase, or combination strategies of *aas* inactivation either with *tesA* or *lipA* overexpression [9, 13–15]. In this study, we created a *Synechocystis* sp. PCC6803 engineered strain with double gene disruptions of *aas* and *sll1951*, encoding S-layer, resulting in significantly increased secreted FFA content under nitrogen deprived conditions.

The hemolysin-like protein (HLP) Sll1951, surface layer protein (S-layer), is the outermost cell component in archaea and bacteria (Fig. 1). Especially, in Gram-negative bacteria including cyanobacteria, the S-layers are closely associated with the lipopolysaccharide on the outer membrane, while some S-layers in archaea are mushroom-like subunits (reviewed in [31]). Recently, several functions of S-layer in cyanobacteria have been addressed including a barrier against the adsorption of some toxic compounds and antibiotics, such as CdCl_2_, CuSO_4_, antibiotics (kanamycin, ampicillin), a component related to mobility in some motile species, a template of natural mineral formation process on surface in some species living in high mineral habitats [32–34]. The *sll1951* deletion mutation in *Synechocystis* 6803 had similar growth rate and carotenoid content to WT cells under photoautotrophic growth condition [21]. This is in agreement with our result under BG_11_ growth condition, strain KS or *Synechocystis* lacking *sll1951*, grew-like WT cells with similar accumulation of both chlorophyll *a* and carotenoids, except higher photosynthetic efficiency (Fig. 3). A S-layer disruption in *Synechocystis* did not generate any severe effects on cell growth and photosynthesis. More strikingly, we observed increased growth of strain KS grown in BG_11_ without NaNO_3_ (BG_11_-N) medium with green colored cell cultures (Fig. 5). As known for cyanobacterial chlorosis process, cells turn blue–green to yellow color during nitrogen deprivation, because phycobilisomes, as well as chlorophyll *a*, are degraded leading to decreased photosynthetic activities [35–37]. Therefore, our observations indicate that the lack of S-layer may enhance the exchange or transport activities of some essential compounds which consequently helps the cells to survive under nitrogen deprived conditions. Although it was previously shown that the *Δsll1951* mutant of *Synechocystis* sp. PCC 6803 may secret high quantities of protein into the medium [22], further experimental data and research are still needed to determine how nitrogen deprivation and S-layer disruption are related. For the other aspect, the strain KS, *Synechocystis* lacking the S-layer protein, may thrive better in lower nitrogen environments, since the production of the S-layer protein certainly consumes substantial amounts of nitrogen. In addition, although we found a lower cell growth under BG_11_ condition of both strains KA, *Synechocystis* lacking *aas* gene, and KAS when compared with WT cells, they all showed similar growth under BG_11_-N condition.

We demonstrate a significant increase of intracellular lipids and FFA secretion in all engineered strains (Table 3). It was worth to note that strain KAS showed the highest capacity to produce total contents of intracellular lipids and secreted FFAs, about 39.0%w/DCW or 178.5 mg/L at day 5, when compared, e.g., with strain KA [9]. Although strain KS secreted a lower level of FFAs, about 11.1%w/DCW or 15.0 mg/L compared to the other engineered strains, a higher FFA secretion was noted when compared to WT cells at days 5 and 10, in agreement with a previous report of a *Δsll1951* strain of *Synechocystis* sp. PCC 6803 with higher FFA secretion [13]. Under BG_11_ growth condition, the *aas* and *sll1951* gene disruptions slightly induced PHB accumulations when compared to that in WT cells but with a more significant increase in glycogen pool size, particularly in strain KA, about 21.4%w/DCW, 5.7 fold increase compared to WT cells (Fig. 7). This suggests that the deletion of *aas*, involved in FFA recycling process, can enhance the glycogen accumulation as carbon storage in *Synechocystis*. This is supported by the lower *glgX* transcript level, related to glycogen degradation, in strain KA compared to that in WT cells (Fig. 8A). For strain KS, the disruption of *sll1951* seemed to stimulate glycogen and membrane lipid degradation, as evidently demonstrated by high *glgX* and *lipA* transcript levels compared to WT cells.

**Fig. 9:**
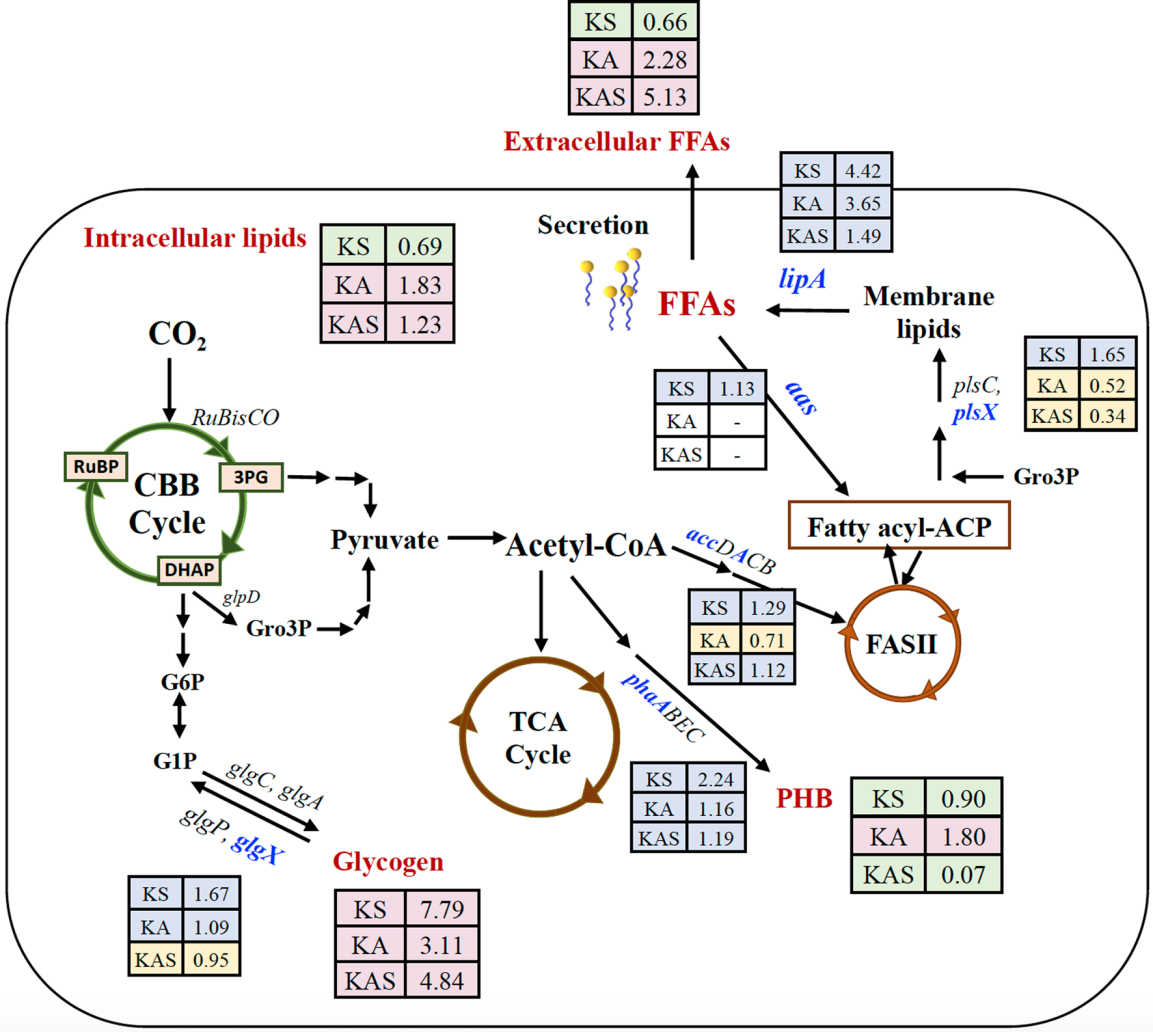
Summary of obtained results, products and gene expression levels, in the engineered strains compared to *Synechocystis* sp. PCC 6803 WT cells after 10 days of growth in BG_11_-N. Each box's number represents the fold of that value divided by WT. When compared to WT, the green and pink colored boxes show lower and higher folds of that product, respectively. For yellow and blue boxes represent lower and higher folds of that transcript amount when compared to WT, respectively

Nutrient (nitrogen) deficiency was addressed in this study to gain more understanding of carbon storage and fatty acid and lipids syntheses by applying BG_11_-half N and BG_11_-N growth conditions for 15 days (Figs. 4 and 5). For the BG_11_-half N condition, increased total contents of intracellular lipids and secreted FFAs were noted in all engineered strains compared to WT cells, particularly in strain KA at day 5 (60.0%w/DCW) and KAS (73.0%w/DCW) at days 5 and 10, respectively (Fig. 6A, B). Reduced nitrogen level (BG_11_-half N) did not significantly affect PHB content, except a lower level in strain KS and a higher content in strain KAS (Fig. 7A). The dramatic increase of glycogen accumulation was apparently induced by the lower nitrate condition employed, especially in strain KA (Fig. 7B). These results may suggest that higher glycogen accumulation contributes to higher growth and intracellular pigment contents under limited nitrogen supply, BG_11_-half N condition (Fig. 4), in agreement with earlier studies on glycogen metabolism under environmental stresses [38, 39]. Moreover, engineered strains, exposed to growth medium lacking nitrate (BG_11_-N), showed higher total levels of intracellular lipids and secreted FFAs, especially in strains KA and KAS, about 63.4 and 70.8%w/DCW, respectively, at day 10 (Fig. 6C). In Fig. 9, the summary of all engineered strains compared with WT was shown under BG_11_-N condition for 10 days. Strain KAS certainly secreted the highest level of FFAs, about 5.1 fold increase in comparison with WT and a 0.07 fold decrease in PHB accumulation (Figs. 6B, 7A and 9). While strain KS responded to N deprivation (BG_11_-N) by maintaining intracellular lipids in similar level to that of WT for 5 days, with enhanced carbon storages of glycogen, about 7.8 fold increase compared to WT, and decreased PHB levels, about 0.9 fold (Figs. 7 and 9). Only *accA* transcript level, involved in the initial step of fatty acid synthesis, was upregulated in KS and KAS strains (Figs. 8B and 9). Since nitrogen is a vital element substantially contributed in biomolecules and cofactors, its deficiency considerably affects cellular mechanisms which force cell coping to this stress for prolonging life by mainly synthesizing energy-containing molecules and increasing carbon or nitrogen source storage, such as glycogen, PHB, and lipid [37, 40–43]. It was noted that the KA strain could cope nitrogen deprivation stress by relatively balancing its carbon storages, lipid and fatty acid syntheses, and FFA secretion (Fig. 9). However, the critical issue for FFA-producing cyanobacteria would result in a rich carbon supply for several other microorganisms. Aseptic production strategy on large scale are, therefore, essential for preventing contamination, and continuous fermentation would offer an appropriate solution.

## Conclusions

Increased levels of FFA secretion were achieved in engineered strains of *Synechocystis* sp. PCC 6803 (KA, KS, and KAS) by affecting the *aas* gene encoding acyl-ACP synthase in FFA recycling and *sll1951* gene encoding surface layer of outer membranes resulting in significant increases of both intracellular lipids and secreted FFAs. Strain KAS with non-functional *aas* and *sll1951*, showed considerably a higher FFA-secreting under both BG_11_ and nitrogen deprived growth conditions (BG_11_-half N and BG_11_-N) with less PHB accumulation. Interestingly, disrupting the S-layer did not affect cell growth, it even improved under nitrogen deficiency conditions. FFA-producing and excreting cyanobacterial cells are promising cell factories for biotechnology applications including biofuel production.

## Materials and methods

### Strains and culture conditions

The host propagation, *Escherichia coli* DH5α strain, was grown either on agar plate or in liquid medium of Luria Bertani (LB) containing 35 µg/mL of kanamycin (Km) and 35 µg/mL of chloramphenicol (Cm) at 37 °C. Cyanobacterium *Synechocystis* sp. PCC 6803 cells were grown in BG_11_ medium on rotary shaker at 28 °C and continuous light illumination of 50 µmol photons m^−2^ s^−1^. Two engineered strains of *Synechocystis* KA (*Δaas*) and KAOL (KA with overexpressing *lipA*) were obtained as described previously [8, 9]. In this study, the *Δsll1951* mutant (KS) and *Δsll1951_Δaas* mutant (KAS) were constructed (Table 1). All strains were cultured in BG_11_ medium containing 35 µg/mL of kanamycin and 35 µg/mL of chloramphenicol.

### Constructions of recombinant plasmids

To construct the recombinant pJSKm plasmid, pJet1.2 blunt end vector was used to insert a kanamycin resistance cassette gene (*km*^*r*^) fragment between *sll1951* sequences. The *sll1951* fragment with its designed size of about 1980 bp was amplified by PCR using a pair of primers; Sll1951_F and Sll1951_R (Table 2). After that, the *sll1951* fragment was introduced into a pJet1.2 vector by blunt end ligation generating a pJetS vector. The antibiotic kanamycin resistance cassette gene (*km*^*r*^) fragment was amplified by PCR using pEERM_Km vector from the previous study as the template [44], and used Km_FKpnI and Km_RKpnI as the primers (Table 2). Both of *km*^*r*^ fragment and pJetS vector were digested with the same restriction *KpnI* enzyme and subsequently ligated by T4 ligase, and generated the recombinant pJSKM plasmid (Table 1).

### Transformation of Synechocystis cells

Two host cells including *Synechocystis* sp. PCC 6803 wild type (WT) and KA strains were grown in BG_11_ medium until an optical density of 0.3–0.5. The cells were harvested by centrifugation at 5000 rpm (2516 ×*g*) for 10 min. The cell pellets were washed by fresh BG_11_ medium and harvested by centrifugation at 5000 rpm (2516 ×*g*) for 10 min. The 1 µg of recombinant plasmids were separately added into condensed WT and KA cells and incubated at 28 °C for 6 h and inverted the tubes every 2 h. Then, the condensed cells were spread on a 0.45 µm sterile nitrocellulose membrane placed over BG_11_ agar plate overnight and then transferred that membrane to place over BG_11_ agar containing 35 µg/mL chloramphenicol or both of 35 µg/mL kanamycin and 35 µg/mL chloramphenicol depending on their host cells. Obtained colonies were collected and examined for gene location and segregation by PCR analysis using specific pairs of primers (Table 2).

### Cell cultivation and nitrogen deficiency treatments

Cell culture with mid-log phase of growth was harvested by centrifugation at 6000 rpm (3622 ×*g*) for 10 min and transferred into various nitrogen deficiency conditions including BG_11_ medium containing 17.6 mM NaNO_3_, BG_11_ medium containing 50% NaNO_3_ concentration (8.8 mM NaNO_3_) or BG_11_-half N, and BG_11_ medium without NaNO_3_ (BG_11_-N). The OD_730_ at beginning of cultivation was about 0.1 and continuously cultured for 15 days.

### Determinations of cell growth and pigment contents

*Synechocystis* cell growth was monitored by a spectrophotometer during cultivation. The pigment contents including chlorophyll *a* (chl *a*) and carotenoid were extracted and determined as described previously [45, 46]. One milliliter of cell culture was harvested and centrifuged at 6,000 rpm (3622 ×*g*) for 10 min. *N,N-*dimethylformamide (DMF) was added into a fraction of cell pellets to extract the pigments. After a quick centrifugation, the pigments in the supernatant were determined by measuring the absorbances (Abs) at 461, 625 and 664 nm using a spectrophotometer, and calculated according to [45, 46]. The results are normalized to cell numbers corresponding to 1.0 × 10^8^ of the cells.

### Measurement of oxygen evolution rate

Five mL of cell culture were centrifuged at 6000 rpm (3622 ×*g*) for 10 min. Cell pellets were resuspended by adding 2 mL of fresh BG_11_ medium and incubated in the darkness for 30 min. After that, the cell suspension was measured for oxygen evolution by Clark-type oxygen electrode (Hansatech instruments, UK) at room temperature (25 °C). The data in terms of the O_2_ evolution rate were presented as µmol/mg chlorophyll *a*/h.

### Lipid extraction

Ten mL of cell culture was harvested by centrifugation at 6000 rpm (3622 ×*g*) for 10 min. Lipids, which are represented as intracellular lipids and extracellular free fatty acids, respectively, were extracted from the cell pellets and supernatant fraction. The lipids were extracted according to the Bligh and Dyer method [47] with slight modification. A glass tube containing cell pellets was filled with 1 mL of a 2:1 chloroform (CHCl_3_): methanol (CH_3_OH) solution, and the supernation fraction was added with a 5 mL solvent solution. The reaction mixture tube was then incubated in a water bath at 37 °C for 2 h. Then, one mL of 0.88% (*v*/*v*) potassium chloride (KCl) was added and vortexed for few seconds. After centrifugation of the reaction mixture tube at 3000 rpm (906 ×*g*) for 5 min, the lower organic phase containing lipids was collected. Then, the chloroform solvent was evaporated at 70 ºC.

### Determinations of total lipid and free fatty acid contents

The total lipid and extracellular free fatty acid contents were determined by potassium dichromate oxidation reaction method [48]. The 0.5 mL of K_2_Cr_2_O_7_ (0.18 M) and sulfuric acid were added into the glass tube of extracted lipids. The reaction mixture was heated at 105ºC for 30 min. After the mixture was cooled down to room temperature, distilled water (0.5 mL) was added before measuring the absorbance at 600 nm (Abs_600_) using spectrophotometer. The canola oil was used as a commercial standard, prepared as same as sample. The unit of lipid content was represented by the percentage ratio of lipids to dry cell weight (%/wDCW). Dry cell weight (DCW) measurement was performed by dehydrating harvested cell pellets in the 60–70 °C oven until obtaining a constant dry weight.

### Determination of PHB contents by HPLC

Five mL of cell culture were harvested by centrifugation at 6000 rpm (3622 ×*g*), 10 min. One hundred µL of adipic acid (20 mg/mL) and 800 µL of concentrated H_2_SO_4_ was added into the tube of cell pellets and further boiled at 100 °C for 1 h for converting of PHB to crotonic acid. After that, 50 µL of the reaction mixture was diluted with 1.20 mL of ultrapure water. Then, one mL of solution was filtered through PP Syringe filter 0.45 microns, 13 mm. and collected in a glass vial for HPLC analysis (Shimadzu HPLC LGE System, Japan). A carbon-18 column with inert sustain 3 µm (GL-Sciences, Japan) was used and performed with a flow rate of 1.0 mL/min. The running buffer was 30% (v/v) acetonitrile in 10 mM KH_2_PO_4_ at pH 2.3. The amount of crotonic acid was detected at 210 nm of UV detector. The commercial standard of crotonic acid was prepared as same as samples. The PHB content is represented as a percentage of PHB per dried cell weight (%w/DCW).

### Determination of glycogen content

One mL of cell culture was harvested by centrifugation at 6000 rpm (3622 ×*g*), 10 min. Cell pellets were collected, and mixed with 600 µL of 30% (*v*/*v*) KOH. The mixture was then heated at 90 °C for 1 h. The supernatant was separated by centrifugation at 12,000 rpm (14,489 ×*g*) for 10 min, then it was transferred into a 1.5 mL microcentrifuge tube. After adding 900 mL of the absolute ethanol into the solution tube, it was incubated at − 20 °C for overnight to precipitate glycogen. The glycogen sediment fraction was harvested by centrifugation at 12,000 rpm (14,489 ×*g*) 4 °C for 10 min, and completely dried at 60 °C for overnight. After that, the sediment was dissolved with one mL of 10% (*v*/*v*) H_2_SO_4_. To determine glycogen content, the dissolved sample (0.5 mL) was taken to mix with 1 mL of anthrone solution (2 g/L anthrone dissolved in concentrated H_2_SO_4_). The reaction mixture was vigorously vortexed, and subsequently heated at 90 °C for 10 min. The sample solution was then measured by spectrophotometer at the absorbance of 625 nm. A commercial glycogen standard (Sigma-Aldrich) was prepared and used for calibrations. The unit of glycogen content presented represents by the percentage of glycogen per the dried cell weight (%w/DCW).

### Reverse transcription polymerase chain reaction

Fifteen mL of cell culture was harvested by centrifugation at 6000 rpm (3622 ×*g*), 10 min, and the total RNA was extracted using 1 mL of TRIzol^®^ Reagent (Invitrogen, Carlsbad, CA, USA). After that, the isolated RNAs were treated with RNaseI-free DNAseI (Fermentas, Life Sciences, Canada) to remove any DNA contaminants and then converted RNAs to cDNA using ReverTra Ace® qPCR RT Master Mix (Toyobo, Osaka, Japan). Then, the cDNA was used as a template for PCR analysis of genes involved in lipid biosynthesis and neighboring pathways including *glgX, phaA, accA, aas, plsX,* and *lipA.* The *16 s* rRNA was used as reference*.* All RT-PCR primers used in this study are listed in Table 2. For PCR condition, it was first started by 98 °C for 3 min, followed by proper cycles of each gene at 98 °C for 15 s, the primer melting temperature (Tm) for 35 s, 68 °C for 15 s to extend the DNA strand, and 68 °C for 5 min at the last step. The cycle numbers and Tm of each primer pair are shown in Table 2. PCR products were verified by electrophoresis on 1.2% (w/v) agarose gels and the intensity of bands was detected using a Syngene Gel Documentation (SYNGENE, Frederick, MD).

## Data Availability

Data generated and analyzed during this study are included in the published article.

## Abbreviations

AAS: Acyl–acyl carrier protein synthetase
ACP: Acyl carrier protein
Car: Carotenoids
Chl *a*: Chlorophyll *a*
CO_2_: Carbon dioxide
DCW: Dry cell weight
DMF: N,N-dimethylformamide
FFA: Free fatty acid
h: Hour
lipA: Lipase A
m: Meter
μg: Microgram
mL: Milliliter
min: Minute
nm: Nanometer
OD: Optical density
PCR: Polymerase chain reaction
plsX: Putative acyltransferase
PHB: Polyhydroxybutyrate
rpm: Revolutions per minute
s: Seconds
S-layer: Surface layer protein
WT: Wild type
